# Obesity: an asthma aggravating factor?

**DOI:** 10.1186/1939-4551-8-S1-A46

**Published:** 2015-04-08

**Authors:** José Elabras Filho, Sérgio Duarte Dortas, Elisabete Da Silva Blanc, Cristiane Fernandes Moreira, Bruno Emanoel Carvalho Oliveira, Ana Carolina Miranda Carvalho Ferreira Fernandes De Sousa, Alfeu Tavares França, Alfeu Tavares França

**Affiliations:** 1 Hospital Universitário Clementino Fraga Filho Hucff-Ufrj, Brazil; 2 Universidade Iguaçu, Brazil; 3 Hospital São Zacharias, Brazil

## Background

Several trials have demonstrated an association between obesity and asthma severity. Body Mass Index (BMI) is a metric used to estimate the amount of body fat. It’s one of the best methods for population assessment of overweight and obesity. We have investigated the relationship between BMI and asthma severity in an adult asthmatic population from the Clinical Immunology outpatient service of an University Hospital.

## Methods

142 adult patients, diagnosed by clinical spirometric findings as asthmatics, were divided in two groups based on their asthma severity: group 1 [(n= 72): Mild Intermittent and Mild Persistent Asthma] and group 2 [(n= 70): Moderate and Severe Persistent Asthma]. Patients BMI were calculated and compared between both groups.

## Results

Patients mean age was 49.5 years-old, most of them were female and caucasian. 26.1% of them were former smokers. Mean weight was 66.17kg in group 1 and 69.36kg in group 2. Mean height were 159.23cm and 157.1cm respectively. Mean BMI was significantly higher (p<0,05) in group 2 (BMI=28,06), comparing to group 1 (BMI=26,15).

## Conclusions

In this study, BMI was associated with asthma severity.

